# Which Therapy for Non-Type(T)2/T2-Low Asthma

**DOI:** 10.3390/jpm12010010

**Published:** 2021-12-23

**Authors:** Fabio L. M. Ricciardolo, Vitina Carriero, Francesca Bertolini

**Affiliations:** Department of Clinical and Biological Sciences, San Luigi Gonzaga University Hospital, University of Turin, Orbassano, 10043 Turin, Italy; vitina.carriero@unito.it (V.C.); francesca.bertolini@unito.it (F.B.)

**Keywords:** non-T2 asthma, endotypes, therapy

## Abstract

Currently, the asthmatic population is divided into Type 2-high and non-Type 2/Type 2-low asthmatics, with 50% of patients belonging to one of the two groups. Differently from T2-high, T2-low asthma has not been clearly defined yet, and the T2-low patients are identified on the basis of the absence or non-predominant expression of T2-high biomarkers. The information about the molecular mechanisms underpinning T2-low asthma is scarce, but researchers have recognized as T2-low endotypes type 1 and type 3 immune response, and remodeling events occurring without inflammatory processes. In addition, the lack of agreed biomarkers reprents a challenge for the research of an effective therapy. The first-choice medication is represented by inhaled corticosteroids despite a low efficacy is reported for/in T2-low patients. However, macrolides and long-acting anti-muscarinic drugs have been recognized as efficacious. In recent years, clinical trials targeting biomarkers playing key roles in T3 and T1 immune pathways, alarmins, and molecules involved in neutrophil recruitment have provided conflicting results probably misleading (or biased) in patients’ selection. However, further studies are warranted to achieve a precise characterization of T2-low asthma with the aim of defining a tailored therapy for each single asthmatic patient.

## 1. Introduction

Currently two main inflammatory phenotypes of asthma are recognized, namely the “Type 2 (T2) high” and “T2-low/non-T2” asthma. Approximately a half of the asthmatic population is affected by non-T2 asthma which includes the neutrophilic, mixed, and paucigranulocytic phenotypes. According to the parameters considered and to their cut-offs, the prevalence of each phenotype can vary. Different studies by means of sputum induction reported that the percentage of patients with neutrophilic (including mixed) and paucigranulocytic ranged between 12–27.6% and 31–47.9%, respectively. Moreover, it has been revealed that the majority of acute asthma exacerbations in adults has a neutrophilic inflammatory background [1]. Since both neutrophilic and paucigranulocytic asthma still lack tailored therapies, their prevalence could suggest the relevance of further research.

Each phenotype is supposed to be underpinned by multiple molecular mechanisms, the endotypes, which are responsible for highly variable symptom manifestations and responses of patients to therapies; this reflects the high heterogeneity and complexity of asthma. De facto asthma is a collection of different airway diseases, with variable and reversible airflow obstruction, characterized by “multiple” triggers, “multiple” inflammatory signals, “multiple” structural alterations and “multiple” clinical presentations.

T2-high asthma is the phenotype whose molecular bases have been better characterized, with a lymphocyte T helper 2 (Th2)-and innate lymphoid cell 2 (ILC2) -driven immune-inflammatory responses.

In contrast, the T2-low endotypes have not been clearly defined yet, but it has been recognized a pivotal role for neutrophils.

In this context, the anti-inflammatory effect of corticosteroids therapy should be considered, as the existing formulations can influence eosinophil apoptosis and prolong neutrophil cell survival [2,3]. Thus, a high dose of corticosteroid treatment may modulate a switch to neutrophilic asthma phenotype [4]. The researchers have identified molecular pathways that could contribute to the pathogenesis of the phenotype and that are represented by: (i) type 1 (T1) immune response driven by interferons (IFN), (ii) type 3 (T3) inflammation mediated by Th17 cytokines, (iii) systemic inflammation associated with IL-6 release and obesity, and (iiii) lack of inflammatory processes resulting in paucigranulocytic phenotype.

## 2. Stratification of T2-Low Asthma Patients

Rather than being based on the identification of hallmarks of the phenotype, the definition of T2-low asthma is supported by the absence of the characteristic inflammatory and immune biomarkers of T2-high asthma, that is, fractional exhaled nitric oxide (F_E_NO) increase, high levels of blood and sputum eosinophils and high levels of serum total IgE. This characterization is accepted and diffused in clinical practice, probably because it uses non-invasive and easy to achieve biomarkers. Overall, by using this approach in a real-world setting, asthmatics with T2-low asthma were reported to be older, with a later age of onset, obese, with lower control, with a higher exacerbation rate, and corticosteroid resistant [5,6,7]. However, this stratification, based on an exclusion strategy, might prove unproductive in the context of personalized medicine, as it fails to provide distinct treatable traits or biomarkers serving as therapeutic targets. Moreover, it is noteworthy that in patients with T2-low, asthma can be detected the presence of T2 biomarkers [8]. This gap is partly filled by the assessment of inflammatory profiles in sputum and biopsy samples. Although these analyses can be performed only in laboratories of highly specialized centers and present a certain degree of invasiveness, they have the advantage of allowing a direct description of airway inflammation. This methodological approach leads to the description of the T2-low asthma airway inflammatory profiles that are defined as neutrophilic (high neutrophil counts), mixed (concomitant presence of high levels of eosinophils and neutrophils), and paucigranulocytic (lack of eosinophilic and neutrophilic infiltration) through the definition of airway inflammatory cut-offs. Recently, a work of Hinks et al. proposed an algorithm to diagnose T2-low asthma in clinical practice that includes both the exclusion of T2-high asthma hallmarks and different approaches, such as sputum cell counts, genetic analysis, and volatile organic compounds measurements [9].

Regarding sputum, several cut-off values have been reported to distinguish between T2-high and T2-low asthma, ranging from 43% to 76% [10,11,12], and this variability rises when it comes to the definition of mixed inflammatory phenotype. Indeed, this inflammatory profile has been defined by sputum percentages ≥ 76% for neutrophils coupled with ≥3% for eosinophils [9] and sputum neutrophil proportion > 61% with an eosinophil proportion > 1.01% [12]. Moreover, it has been shown that sputum eosinophil and neutrophil concentrations can vary over time as a consequence of environmental stimuli [13,14].

**Figure 1:**
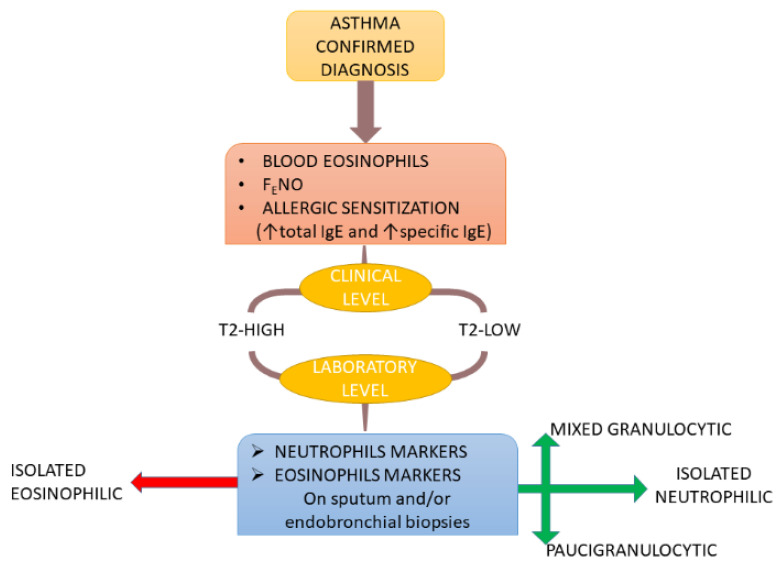
Algorithm summarizing the steps leading to clinical and biological characterization of T2-high and T2-low phenotypes. Modified from Hinks et al. [9].

Bronchial biopsy can be considered as the gold standard analysis to describe the airway inflammatory process, although reliable cut-off points still lack. Wenzel was the first author to characterize neutrophilic severe asthma on the basis of inflammatory cell expression in the lung biopsies of asthmatic patients, and stratified severe asthmatics in eosinophil (+) and eosinophil (−), depending on bronchial eosinophil numbers [15]. Recently, Ricciardolo et al. distinguished patients with neutrophilic asthma from those with eosinophilic asthma on the basis of the submucosal neutrophil and eosinophil thresholds (bronchial neutrophilia ≥ 47.17 cells/mm^2^ and bronchial eosinophilia ≥ 12.45 cells/mm^2^) [16]. In addition, Bullone et al. distinguished between neutrophilic asthma phenotype (≥ 47.17 cells/mm^2^) and high neutrophilic asthma phenotype (≥94.34 cells/mm^2^) [17]. Figure 1 shows the biomarkers that are used in clinical and laboratory practice in order to characterize T2-high and T2-low asthma. The different methodologies used to define asthma phenotypes, if taken separately, could provide a confounded observation; however, a synergic application could lead clinicians to a better understanding of asthma. The efforts toward asthma characterization resulted in the definition of molecular mechanisms likely driving T2 low asthma.

## 3. Mechanisms of T2-Low Asthma

T2-low asthma is strongly associated with neutrophilic airway inflammation. Neutrophilic asthma has a lower incidence than eosinophilic or mixed phenotypes, but can manifest itself as a severe corticosteroid-resistant form of the disease [18]. Other characteristics that frequently associate with severe neutrophilic asthma are older age, impaired lung function, less bronchodilator reversibility, microbial infections, cigarette smoking, and obesity [19,20]. Neutrophils represent 50–70% of human circulating leukocytes, and are a key component of the innate immunity. They are produced and reside as granulocyte–monocyte progenitors in the bone marrow, from which they are released into the blood to rapidly reach the site of infection or inflammation. In blood, these leukocytes are present as mature and immature neutrophils [21], the latter being involved in asthma pathogenesis. Researchers pointed out the heterogeneity existing in the neutrophil population and have identified a low-density phenotype consisting of both immature and activated mature neutrophils, additional to the high-density ones, and associated with cancer and chronic inflammatory diseases, such as asthma [21,22]. Furthermore, Uddin and colleagues showed that in the sputum of asthmatic patients, neutrophils had an anti-apoptotic activity greater than that in healthy subjects, and that this characteristic increased when asthma severity was raised [23].

**Figure 2:**
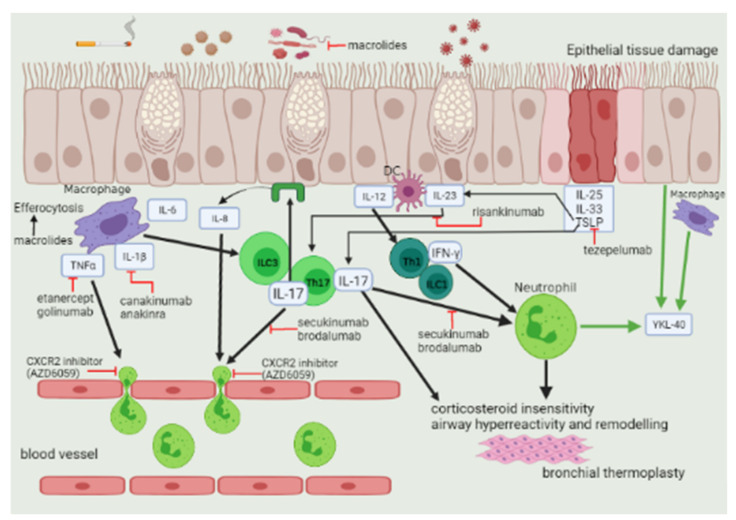
Neutrophilic inflammation process and associated therapeutic mechanisms of action. Allergens, pollutants, cigarette smoke, viruses, and bacteria can damage and stimulate the airway epithelium which releases alarmins (TSLP, IL-33, IL-25) and chemokines such as IL-8 acting as neutrophils chemoattractant and activator by binding CXCR2 receptor. Macrophages and dendritic cells (DC) elicit the recruitment of neutrophils and the release of pro-inflammatory cytokines by Th17/ILC3 and Th1/ILC1 cells. Bronchial thermoplasty impacts airway remodeling, resulting in a reduction of airway smooth muscle mass and an improvement of asthma control. Black arrow, induction/activation; green arrow, release; and red line, inhibition. Created in BioRender.com 30 October 2021.

The molecular mechanisms underlying neutrophilic inflammation of the airways have not been clarified yet, but two main agreed processes are Th1 and Th17 immune responses (Figure 2) [24,25].

### 3.1. Type 1 Immunity

Type 1 (T1) immune processes occur following the activation of pattern recognition receptors, such as toll-like receptors (TLRs), in response to microbial infections and involve immune cells able to release IFN-γ, which are CD4+ Th1 cells, type 1 ILCs (ILC1), CD8+ T cells, and natural killer T cells (NK) [26,27,28]. After the interaction between TLRs and microorganisms, dendritic cells (DCs) produce interleukin (IL)-12, which is able to promote the differentiation of Th naive cells in Th1 through the activation of signal transducer and activator of transcription 4 (STAT 4) [26]. This leads to the induction of T-bet, which is the key transcription factor of the Th1 cell. The polarization towards Th1 phenotype is also induced by IFN-γ that modulates the STAT1/T-bet pathway. Furthermore, IFN-γ can activate mononuclear phagocytes (MPs) and promotes the recruitment of Th1. Findings regarding the pathogenic role of T1 pathways in asthma are controversial. On one hand, it has been shown that asthmatic patients more susceptible to viral infections have a deficiency of IFN-γ signaling in their epithelium; on the other hand, severe forms of asthma were associated with higher expression of IFN-γ and IL-17A in the airways. Moreover, IFN-γ along with CXCL10 is involved in corticosteroid resistance [29].

### 3.2. Type 3 Immunity

T3 immunity is mediated by the IL-17 cytokine family members, with a predominant involvement of IL-17A and IL-17F, which induce the epithelial cells and fibroblasts to release CXCL1 and CXCL8 (IL-8) chemokines responsible for neutrophil recruitment in the airways. In addition, IL-17 cytokines can promote activation and migration of neutrophils [26,30,31] and are produced by a plethora of cells including Th17, NK, CD8+ T cells, γδ T cells, ILC1, and ILC3, which were reported to be present at high levels in the blood, sputum, and bronchial biopsies samples of severe asthmatic patients [28,32]. The cytokines TGF-β, IL-6, IL-1β, and IL-23 drive the differentiation of Th17 cells whose secretory activity (IL-17A/F and IL-22) results in steroid resistance, secretion of epithelial-derived chemokines responsible for neutrophil recruitment to the airway, mucous cell metaplasia, airway smooth muscle mass hyperplasia, and fibroblast proliferation (IL-17A) [28,33,34]. Recent evidence supports the concomitant occurrence of T2 and T3 immunity in asthmatics with the presence of Th2/Th17 cells in blood and bronchoalveolar lavage of stable allergic and severe asthma patients [35,36]. The plasticity of Th17 cells has been also demonstrated; they can shift towards a Th2 phenotype producing T2 cytokines [37]. In line with these observations, our research group has recently reported elevated levels of serum IgE in the bronchi of asthmatics, characterized by high neutrophilia [16]. These findings, together with the study of Choy et al., reporting that Th17 response can be promoted by suppressing the T2 pathway, suggest that a double blockade of T2 and T3 processes may be beneficial in asthma [38].

Studies conducted on murine models and asthmatic patients showed that Th17 immune response plays a role in the airway remodeling [39]. In particular, Lu and colleagues reported that angiogenesis induced during a prolonged allergen exposure is mediated by Th17 cells, with IL-17A acting as a key mediator of vascular remodeling in the airways [40]. Findings supporting the Th17-promoted angiogenesis are: (i) CXCL1 and CXCL2 are involved in the recruitment of endothelial progenitor cells forming new vessels; (ii) IL-1β stimulate epithelial cells, fibroblasts, and smooth muscle cells to release the proangiogenic vascular endothelial growth factor (VEGF); and (iii) IL-8 is a potent elicitor of endothelial cell proliferation, chemotaxis, and survival, along with activation of metalloproteases [30,39,41]. In asthma, increased vascularity is associated with higher severity [30,42,43] and this observation is in line with previous works revealing that the airways of severe asthmatics express higher levels of VEGF-A than those of mild asthmatics and healthy individuals [44,45]. Other mechanisms that could be involved in T2-low asthma are those associated with IL-6 and obesity.

## 4. IL-6 and Obesity

The existence of obesity-induced asthma, prevalently in late-onset, asthmatic females has been demonstrated and linked to neutrophilic and paucigranulocytic airway inflammation [46,47,48]. The hypothesized mechanism causing this phenotype of asthma is a macrophage-dependent inflammation of the adipose tissue resulting in a pronounced release of IL-6, TNF-α, and leptin that can be reversed with a weight loss [49]. Recent studies have shown a link between neutrophilic inflammation and airway hyperresponsiveness associated with obesity, with the involvement of ILC3 producing IL-17 [50].

IL-6, released by macrophages in the adipose tissue, is a cytokine that has recently gained attention as a key player in non-T2 asthma. This is a pleiotropic cytokine, which exerts both pro- and anti-inflammatory activities and whose levels have been found elevated in patients presenting neutrophilic asthma, especially associated with obesity [51]. The pro-inflammatory activities of IL-6 are involved in the acute phase response to infection inducing systemically C reaction protein, Complement component C3, Fibrinogen, and serum amyloid A protein [52].

The exact pathogenic role of IL-6 in this phenotype of asthma has not yet been defined. However, in vitro studies showed the possible IL-6 contribution in smooth muscle cell proliferation and airway remodeling. In addition, IL-6, together with TGFβ, has been proved to induce Th17 differentiation [20]. Moreover, preclinical studies on mice suggested a cooperation between IL-6 and IFN-γ during inflammation. IL-6 and IFN-γ could both contribute to emphasize pro-inflammatory cascade likely through the mediation of Interferon Regulatory Factor 1 (IRF1). Additionally, the interplay between IL-6 and IFN-γ could regulate the trafficking and clearance of neutrophils in acute inflammatory status [53,54]. Overall, these paramount observations highlight the interplays occurring within different T2-low asthma inflammatory processes. A peculiar phenotype of non-T2 asthma is paucigranulocytic asthma which is defined by the absence of a specific inflammatory signature.

## 5. Paucigranulocytic Asthma

Paucigranulocytic asthma (PGA) is considered as an independent phenotype of stable asthma [55] since PGA patients show better lung function and lower incidence of severe refractory asthma compared to the other phenotypes [56].

PGA is characterized by airway eosinophil and neutrophil expressions below the above-cited sputum cut-offs. In fact, in this phenotype of asthma, a disassociation between inflammation and remodeling occurs; there is evidence of structural alterations occurring in the airways of patients with PGA, resulting in a thickening of the subepithelial reticular basement membrane, airway obstruction, and airway hyperresponsiveness (AHR). In conjunction with these structural changes, an increase in TGFβ expression in PGA patients has been reported [57,58,59]. Studies on murine models suggested that AHR could be associated with a modification of airway smooth muscle contractile properties, likely mediated by altered neuronal stimuli [1]. Furthermore, studies on asthmatics patients indicated oxidative stress as a process implicated in the pathogenesis of PGA. Higher levels of glutaredoxin1, an antioxidant enzyme, were detected in the sputum supernatants of patients with PGA [60]. Recent evidence shows the association between PGA and oxidative phosphorylation occurring in bronchial epithelial cells [61]. Another potential cause of PGA is occupational exposure to chemicals such as Toluene diisocyanate (TDI), whose avoidance resulted in a decrease in reticular basement membrane thickness, subepithelial fibroblasts, mast cells, and lymphocytes [62].

The above-cited evidence reveals the high heterogeneity of non-T2 asthma which represents a therapeutic challenge and implies a key role of molecular biomarkers.

## 6. Non-T2 Biomarkers

The assessment of biomarkers in asthma is performed mainly in the sputum, peripheral blood, exhaled air, bronchoalveolar lavage (BAL) fluid, and bronchial biopsies [63].

The scarce knowledge of the endotypes supporting T2-low asthma led to a paucity of agreed biomarkers. Thus, the classification of patients with non-type 2 phenotype is based on the absence or non-predominant expression of T2 markers together with a small number of non-validated biomarkers including sputum neutrophils, blood neutrophils, bronchial neutrophils, IL-17, and IL-6.

Recent evidence showed that high numbers of blood neutrophils are associated with poor asthma symptom control and can predict a greater risk of frequent exacerbations [64,65]. However, GINA guidelines do not recommend blood neutrophilia for asthma diagnosis. In line with this recommendation, Agache et al. observed that blood neutrophils cannot be considered a suitable biomarker for asthma characterization [66]. Findings coming from the assessment of the chitinase-like protein YKL-40 in the blood are opposite to this, as elevated levels of YKL-40 correlated with blood neutrophil counts in children with treatment-resistant asthma and with blood eosinophils in adult asthmatics [67]. Moreover, a recent study performed cluster analysis and identified a subset of asthmatic patients characterized by neutrophilic inflammation with high expression of YKL-40 and poor lung function [68]. In sputum, higher neutrophilia (neutrophil percentage > 61%) was detected in patients with moderate-to-severe asthma and was shown to be in association with lower FEV_1_ [12,69]. Recent works revealed that neutrophilic inflammation was connected with the expression of specific micro RNAs, including miR-233-3p, and with worse lung functionality and quality of life [70,71]. Another biomarker that is involved in neutrophilic inflammation of the airways is IL-8 that is present at higher concentrations (protein and mRNA) in the sputum of neutrophilic asthmatics [72]. Research highlighted the involvement of inflammasome in T2-low asthma. Inflammasomes are multiprotein signaling complexes, with the nucleotide-binding oligomerization domain-like receptor pyrin domain-containing 3 (NLRP3) inflammasome being the most studied and implicated in inflammatory diseases such as neutrophilic asthma [73]. Inflammasome activation is an amplification of the inflammatory process that can be induced through the interaction between various pathogen-associated molecular patterns (PAMPs) or damage-associated molecular patterns (DAMPs) and pattern recognition receptors such as Toll-like receptors (TLRs); or through cytokines such as tumor necrosis factor (TNF) and IL-1β. The consequence of NLRP3 inflammasome activation is the upregulation of NLRP3, caspase 1, IL-1β, and IL-18 expressions [74]. Recent studies reported that patients with neutrophilic asthma had higher sputum expression of NLRP3, IL-1β, and caspase-1 compared to eosinophilic asthma and that upregulated IL-1β signaling in neutrophilic asthma was linked to NLRP3 activation [73].

Recent measurements performed on biopsy specimens allowed the determination of a cut-off (47.17 cells/mm^2^) that could help to characterize neutrophilic asthmatic patients and revealed that highly neutrophilic asthma (94.34 cells/mm^2^) was characterized by higher expression of IL-17F, IL-17A, and IL-22 [17]. Ricciardolo et al. reported higher expression of IL-17F in the lamina propria of asthmatics with severe asthma in conjunction with neutrophilic infiltrate and higher rate of exacerbation [16]. Interestingly, higher expression of IL-17 has been found in severe allergic asthma [75], likely due to a shift from a predominant Th2 immune signature to a mixed Th2/Th17 response [17].

The identification of specific biomarkers allowing the definition of personalized therapy represents a sound future perspective.

## 7. Therapeutic Strategy

### 7.1. Non-Pharmacological Intervention

At present, there is no defined personalized therapy for T2-low asthma and it is necessary to find novel treatments that will have a favorable impact on these patients. An important, but often neglected, intervention is the non-pharmacological treatment which has a low cost and could dramatically improve asthma control of patients.

The non-pharmacological intervention consists of the possible removal of the exposure to environmental/occupational pollutant agents or smoking cessation [28,32,76] and the changes in daily habits.

Recently, Dumas et al. highlighted a strong association between the neutrophilic inflammatory process, oxidative stress, and airway damage in the pathogenesis of irritant-induced asthma [77]. In line with this association, another study showed that a reduction of neutrophilic inflammation and improvement in asthma symptoms was observed after cessation of exposure to irritant agents [78]. Exposure to a plethora of environmental factors, such as smoking, may trigger neutrophilic asthma. It was recently demonstrated by a work that found increased levels of IL-17A and neutrophils in bronchial biopsies of smoking asthmatics compared with the non-smoking asthmatic group [79]. Previously, a clinical trial conducted on 32 asthmatic patients revealed that smoking cessation provoked a significant improvement in FEV_1_ in conjunction with a reduction in sputum neutrophils in asthmatics than those who continued to smoke [80]. These findings suggested that this non-pharmacological intervention could be a possible strategy to reduce neutrophilic inflammation.

Another strategy is represented by a low-fat diet. A work by Li et al. revealed that the levels of some genes, supposed to drive neutrophilic mechanisms, were increased in asthmatic patients after high-calorie and high-fat meals [81]. Patients that follow a high-fat diet compared with the low-fat diet, displayed higher levels of sputum neutrophils and toll-like receptor 4 (TLR4) mRNA expression associated with an impairment of bronchodilator response [82]. Given the high prevalence of obese asthmatics in T2-low asthma in association with the state above, findings suggested that exercise and modifying dietary fat intake may be useful in the therapeutic scheme of these patients. Furthermore, recent evidence demonstrated that airway responsiveness, lung function, quality of life, and asthma severity/control markedly improved with weight loss following bariatric surgery, thus, suggesting that this procedure can be beneficial for asthma in patients with morbid obesity [83,84].

### 7.2. Bronchial Thermoplasty

Innovative treatments (bronchial thermoplasty, BT) have been used recently as therapy for severe uncontrolled asthma, such as paucigranulocytic. BT targets primarily airway remodeling by applying localized radiofrequency, resulting in ablation of the bronchial mucosa [9]. Recent studies revealed that the nerve ablation, reduction of smooth muscle mass area, and RBM thickness induced by BT improves asthma control and quality of life, and reduces hospitalizations and severe exacerbations. Furthermore, evidence suggested that these benefits on asthma control and safety persist 27–48 months following BT treatment [85,86,87]. In the literature, there were some clinical trials regarding the efficacy and safety of BT. In a randomized, controlled trial (AIR trial) 112 moderate or severe persistent asthmatics were enrolled [88]. Patients in the BT group had a reduction of exacerbation rate, and an improvement in the morning peak expiratory flow and quality of life, but no differences were observed for FEV_1_ and airway responsiveness values, compared to the control group [88]. Another clinical trial (RISA trial) evaluated the efficacy of BT in symptomatic severe asthmatics treated with high doses of ICS and LABA [89]. At 52 weeks after BT treatment, asthmatics showed significant improvement in rescue medication use, pre-bronchodilator FEV_1_% predicted, and Asthma Control Questionnaire scores than the control group [88]. Castro and co-workers conducted a Multicenter, Randomized, Double-Blind, Sham-Controlled Clinical Trial (AIR2) on 288 asthmatic patients randomized to BT and sham control who underwent three bronchoscopy procedures [90]. Patients treated with BT showed an improvement in AQLQ score and severe exacerbations rate. The authors concluded that BT provided a novel procedure for the clinician to decrease the morbidity of severe asthmatic patients [90]. Different hypotheses were postulated regarding the mechanisms underlying the BT process involving alteration to the structure or function of airway smooth muscle. The recent TASMA study demonstrated that asthmatic patients, randomized to either bronchial thermoplasty or delayed treatment after six months, showed a reduction in ASM mass after BT when compared to an appropriate non-BT-treated control group [91].

### 7.3. Macrolides

There is preclinical evidence (in vitro studies) that supports the off-label use of macrolides normally prescribed for conditions other than asthma due to their anti-inflammatory effects. Macrolides are often administered in asthma patients, although long-term use could increase the adverse events and the development of microbial resistance [76]. Several studies concur that the administration of macrolide antibiotics in asthma had steroid-sparing effects and reduced exacerbations. The main macrolide studied in asthma is azithromycin, which may act primarily as an antibacterial [9]. In a randomized double-blind placebo-controlled parallel-group multicentre study (AZISAST), the authors randomized 109 exacerbation-prone severe asthmatics with azithromycin (250 mg) or placebo. In subjects with severe non-eosinophilic asthma, azithromycin treatment reduced the rate of severe exacerbations [92]. In a further randomized, double-blind, placebo-controlled trial (AMAZES), 420 adult patients with moderate-to-severe persistent symptomatic asthma were randomized (1:1) with oral azithromycin 500 mg or placebo for 48 weeks, despite maintenance therapy with an inhaled corticosteroid and a LABA [93]. The authors observed that patients in the azithromycin group had a reduction in severe exacerbations and a significant amelioration in asthma-related quality of life. In conclusion, these clinical trials highlighted that the treatment with azithromycin, as add-on therapy, improved quality of life and was well tolerated in the asthmatic population. Despite these promising results and the recommendation of this drug in the current ERS/ATS and GINA guidelines for selected persistently symptomatic adults with severe asthma, there are still many debates about its use. This is due to the adverse effects that macrolides could provoke in the patients, such as diarrhea, QT prolongation, and the promotion of antimicrobial resistance [94]. It has been postulated that the improvements elicited by macrolides and azithromycin could derive from their prompting effect on efferocytosis [95].

### 7.4. Long-Acting Muscarinic Antagonists (LAMA)

Another approach to treat non-T2 asthma is represented by the addition to the conventional therapy of LAMAs, especially tiotropium. The latter has been recently included as an add-on asthma treatment strategy for severe asthmatics (GINA step 4-5) with a history of exacerbations [96]. In 2008, a study performed on 17 patients with severe persistent asthma revealed that after 4 weeks of administration, tiotropium significantly improved lung function (ΔFEV_1_). Furthermore, the authors observed that there was an inverse correlation between the percentage of sputum eosinophils and ΔFEV_1_, but a positive association with the proportion of neutrophils, thus, suggesting that a non-eosinophilic sputum profile is associated with a better response to tiotropium [97]. Casale et al. examined the responses to tiotropium in adult patients with severe and moderate asthma stratifying into T2-high and T2-low groups according to serum IgE levels and blood eosinophil counts. The data revealed that tiotropium when administered concomitant with ICS once daily improved both lung function and asthma control and reduced exacerbations and asthma worsening risks in asthmatics independently of T2 inflammatory status [98].

### 7.5. Biological Agents

Despite the increasing importance obtained in the most recent years, fewer drugs’ development has been evaluated for non-T2 asthma compared to the plethora of biologics identified for T2-high asthma. The success of the personalized therapy for non-type 2 asthma lies in the identification and targeting of specific biomarkers and pathways mediating this phenotype manifestation. At present, some potential targets for therapeutic interventions are available in the literature.

Thymic stromal lymphopoietin (TSLP) is involved in the onset and persistence of inflammation in the airways, mediated by polluting agents and viruses, acting as a regulator of the eosinophilic and neutrophilic response [99]. Airway TSLP is overexpressed in severe asthma, and it has been associated with steroid resistance of airway ILC2 in severe asthma. In human beings, available evidence shows that TSLP provokes the release of IL-23 from dendritic cells, resulting in the differentiation of CD4^+^ cells in Th17 leukocytes [100].

Tezepelumab is a fully human monoclonal IgG2λ antibody that specifically ligates thymic stromal lymphopoietin impeding the human TSLP–TSLP receptor interaction [101]. Tezepelumab was used as add-on therapy for patients with severe uncontrolled asthma due to its safety, tolerability, and efficacy. Several trials have been evaluating the long-term safety and the efficacy of tezepelumab in severe uncontrolled asthmatics [102], as well as assessing the possible use of this drug as a therapeutic strategy in T2-low neutrophilic asthma [101]. In a randomized, double-blind, placebo-controlled trial (PATHWAY Study), Corren et al. estimated the effects of tezepelumab (70 mg, 210 mg, or 280 mg) every 4 weeks, randomizing 584 moderate-to-severe poorly controlled asthmatics. Patients treated with tezepelumab showed a significant reduction in annualized exacerbation rates compared to placebo, independently of their blood eosinophils counts, F_E_NO levels or Th2 status [103]. This evidence supports the contribution of TSLP to the pathophysiology of this difficult-to-treat endotype through the inhibition of several downstream pathways.

The IL-17F and A are cytokines produced predominantly by innate and adaptive lymphocytes and promoting neutrophilic inflammation and steroid resistance in vitro and in vivo [33]. Available evidence reports that higher IL-17F expression in the bronchial mucosa of severe asthmatics is linked to a higher rate of exacerbation and neutrophilic infiltrate [16]. Concerning asthma therapy through the inhibition of IL-17A, two humanized monoclonal antibodies with different mechanisms of action are currently considered. Secukinumab is a human monoclonal antibody that selectively targets and neutralizes interleukin-17A. In a preclinical study on murine models with an elevated number of airway neutrophils, the administration of anti-murine interleukin-17A monoclonal antibody did not induce a reduction in the airway neutrophils from baseline [104]. In a subsequent phase 2 study conducted on patients with uncontrolled asthma, secukinumab did not improve the asthma control, measured through the ACQ score [105]. These less-than-encouraging results led to abandonment of this strategy as a possible asthma treatment. Brodalumab is a human monoclonal antibody that blocks the biologic activity of IL-17A, IL-17F, IL-17A/F heterodimer, and IL-25. The efficacy of brodalumab was assessed in a clinical trial in which uncontrolled asthmatic patients regularly treated with ICS were enrolled. The authors observed that treatment did not induce a significant difference in the ACQ score, asthma symptoms, or lung function, although patients weren’t selected according to airway neutrophilia or IL-17 levels [106]. Similar findings were observed after the treatment with risankizumab, an anti-IL-23 antibody, which blocks Th17 cell differentiation. The severe asthmatics that were included in this ongoing clinical trial, disregarding their cytokine levels or airway neutrophil numbers, showed a worsening in asthma control [107].

Another important cytokine involved in neutrophilic asthma is tumor necrosis factor α (TNF-α), secreted by lymphocytes, mast cells, and macrophages, promoting bronchial hyperresponsiveness and sputum neutrophilia in severe asthmatics [96]. Studies conducted on severe asthmatics treated with soluble TNF receptor (etanercept) revealed an amelioration of bronchodilator responsiveness and a reduction in hyperresponsiveness [108,109]. Unfortunately, in a larger clinical phase 2 trial, golimumab, an anti-TNF, reduced asthma exacerbation risk in severe persistent asthmatics, did not improve asthma control and lung function, and actually provoked serious infections, such as pneumonia, and malignancies [110]. Another possible target is represented by TL1A, a TNF superfamily member that amplifies the Th1 and Th17 responses, suggesting that its inhibition could be efficacious as a therapeutic strategy for non-T2 asthma [9].

Other cytokines involved in neutrophilic inflammation could become reliable targets for the development of biological drugs for non-type 2 asthma.

The inhibition of IL-1β in type 2-low neutrophilic asthma was supported by murine and human studies. In a murine model of severe, steroid-resistant asthma, the administration of either a neutralizing anti-IL-1β antibody or a pharmacological NLRP3 inhibitor (MCC950) suppressed lung IL-1β production and neutrophilic airway inflammation [111]. Monoclonal antibodies that block IL-1β, canakinumab, or block the soluble IL-1 receptor, anakinra, represented two biological drugs potentially useful as personalized medicine approach in the treatment of asthmatic characterized by a neutrophilic airway inflammation [9].

The literature provides more information about the key role that IL-8 plays in neutrophil recruitment and activation; thus, treatments that block binding of IL-8 with CXCR2 have been considered. In an exploratory, single center, open-label, non-controlled, pilot study the treatment with AZD6059, a selective small-molecule antagonist of the human CXCR2 chemokine receptors, on moderate persistent neutrophilic asthmatics (sputum neutrophil > 50%) for 4 weeks, induced the reduction of bronchial (assessed by sputum and biopsy samples) and systemic neutrophils [112]. This work shed light on the pivotal role of CXCR2 on neutrophilic lung tissue infiltration. The findings reported by other studies on severe uncontrolled asthma characterized by neutrophilic inflammation were promising and suggested this signaling as a possible target to this endotype of asthma, although the inhibition of this molecule did not improve the standard clinical outcomes and exacerbation rates [113,114]. The biomarker of systemic inflammation, metabolic dysfunction, and obesity is IL-6 and its sputum and serum levels were increased in non-T2 asthma. The importance of this cytokine in severe asthma will need confirmation by trials inhibiting IL-6 signalling [9].

Figure 2 summarizes the molecular targets of the current therapies.

By and large, the high complexity of T2-low asthma leads to heterogeneous and partially successful therapeutic approaches.

## 8. Conclusions

Notwithstanding the several efforts and the obtained results, the research in the respiratory field has not achieved a clear characterization of non-T2 asthma yet. It should be taken into account that in this phenotype, a complex interplay between different immune-inflammatory pathways can occur, including Th1 and Th17 inflammation, or a combination of Th2 and Th17 inflammation. The majority of asthmatic patients affected by T2-low asthma may exhibit biomarkers belonging to both T2 and T3/T1 immune pathways and the extent of the contribution of each mechanism is hard to determine. On the other hand, the airways of T2-low asthmatic patients can reveal low/absent levels of both T2 and T3/T1 biomarkers. In this perspective, we think that “T2-low asthma” is a more appropriate definition of this phenotype that encompasses paucigranulocytic and neutrophilic (including mixed) asthma. Honestly, it is hard to imagine asthma as a rigid dichotomous disease (T2-high or T2-low), but it probably represents a dynamic spectrum of different mechanisms (sometimes with low or high degree of overlapping) due to various immune-inflammatory and remodeling responses upon different environmental stimuli with predominant pathways variable over time on the basis of the host/airway’s susceptibility and the natural history. Over the past decades, clinical trials led to conflicting results without providing sound evidence supporting the development of personalized therapy in T2-low asthma. Further investigations are eagerly requested to find out suitable molecular targets and prognostic biomarkers that, along with the already recognized TSLP and IL-17, allow to obtain a more precise phenotyping.

## Data Availability

Not applicable.
